# Trends and Disparities in the Use of Immunotherapy for Triple-Negative Breast Cancer in the US

**DOI:** 10.1001/jamanetworkopen.2024.60243

**Published:** 2025-02-03

**Authors:** Jincong Q. Freeman, Dezheng Huo, Sarah P. Shubeck, Nan Chen, Sudha R. Yarlagadda, Rita Nanda, Frederick M. Howard

**Affiliations:** Department of Public Health Sciences, The University of Chicago, Chicago, Illinois; Cancer Prevention and Control Program, UChicago Medicine Comprehensive Cancer Center, Chicago, Illinois; Center for Health and the Social Sciences, The University of Chicago, Chicago, Illinois; Department of Public Health Sciences, The University of Chicago, Chicago, Illinois; Center for Clinical Cancer Genetics & Global Health, The University of Chicago, Chicago, Illinois; Department of Surgery, The University of Chicago Medicine, Chicago, Illinois; Section of Hematology & Oncology, Department of Medicine, The University of Chicago, Chicago, Illinois; Section of Hematology & Oncology, Department of Medicine, The University of Chicago, Chicago, Illinois; Section of Hematology & Oncology, Department of Medicine, The University of Chicago, Chicago, Illinois; Section of Hematology & Oncology, Department of Medicine, The University of Chicago, Chicago, Illinois

## Abstract

**IMPORTANCE:**

Triple-negative breast cancer (TNBC) disproportionately affects Black women. Immunotherapy improves outcomes in early-stage TNBC (esTNBC) and metastatic TNBC (mTNBC). However, racial and ethnic disparities in immunotherapy receipt and its potential association with oncologic outcomes are unknown.

**OBJECTIVE:**

To examine trends and racial and ethnic disparities in immunotherapy receipt and differences in pathologic complete response (pCR) and overall survival (OS) in TNBC.

**DESIGN, SETTING, AND PARTICIPANTS:**

This retrospective cohort study analyzed the 2019–2021 National Cancer Database. Data were analyzed from April 1 to August 31, 2024. The esTNBC cohort included patients with stage II to III TNBC treated with neoadjuvant therapy in 2021. The mTNBC cohort included patients with stage IV TNBC treated from 2019 to 2021, with all patients having received chemotherapy with or without immunotherapy.

**MAIN OUTCOMES AND MEASURES:**

Immunotherapy use, pCR, and OS with respect to race and ethnicity.

**RESULTS:**

A total of 10 724 patients with TNBC were included (mean [SD] age, 56.1 [13.7] years; 473 [4.4%] Asian or Pacific Islander, 2569 [24.0%] Black, 981 [9.1%] Hispanic, 6465 [60.3%] White, and 236 [2.2%] other). Immunotherapy use increased from 5.5% in 2017 to 38.8% in 2021 for mTNBC and from 4.2% in 2017 to 48.0% in 2021 for esTNBC. Of 7655 cases of esTNBC diagnosed in 2021, immunotherapy use was lower in Black patients (788 of 1715 [45.9%]), but this difference was not significant after adjusting for insurance status, treatment facility type, and other key variables. Among patients with esTNBC receiving immunotherapy, pCR was similar by race and ethnicity. Of 3069 cases of mTNBC diagnosed from 2019 to 2021, immunotherapy use was higher in Asian or Pacific Islander patients (36 of 97 [37.1%]) and lower in Black patients (238 of 848 [28.1%]). Black patients had lower odds of immunotherapy receipt than White patients (adjusted odds ratio, 0.63; 95% CI, 0.49–0.80) even after controlling for confounders. Among patients receiving immunotherapy, OS was similar between Black and White patients (adjusted hazard ratio, 0.92; 95% CI, 0.64–1.32).

**CONCLUSIONS AND RELEVANCE:**

In this cohort study of TNBC, Black patients with mTNBC were less likely to have received immunotherapy, even after controlling for socioeconomic factors. In both esTNBC and mTNBC cohorts, patients who received immunotherapy attained similar outcomes across racial and ethnic groups; thus, efforts should be made to ensure equal access to immunotherapy.

## Introduction

Triple-negative breast cancer (TNBC), accounting for 10% to 15% of all breast cancers, is an aggressive subtype that disproportionately affects Black women in the US,^1^ contributing to higher breast cancer mortality rates in Black women.^1,2^ Guidelines recommend systemic therapy for most early-stage TNBC (esTNBC) cases, with neoadjuvant chemotherapy (NACT) recommended for stage II to III tumors.^3^ Pathologic complete response (pCR) to NACT is a key surrogate marker for treatment and long-term survival outcomes. Research has indicated that Black patients with esTNBC are less likely to achieve pCR and more likely than White patients to experience poorer survival outcomes after NACT.^4–6^ In a National Cancer Database (NCDB) study, Black patients with esTNBC diagnosed from 2010 to 2017 had a 15% lower odds of achieving pCR after NACT than White patients, and Black patients had a higher risk of all-cause mortality post NACT.^4^

Immunotherapy has been shown to improve overall survival (OS) for patients with metastatic TNBC (mTNBC) treated with chemotherapy based on the results from the KEYNOTE-355 trial^7^ and has been increasingly used since the accelerated approval of atezolizumab for programmed death-ligand 1 (PD-L1)–positive mTNBC in March 2019.^8^ For esTNBC, randomized data from the KEYNOTE-522 trial have demonstrated a significant improvement in pCR rate (63.1% with immunotherapy and NACT vs 55.6% with NACT alone) and OS.^9,10^ However, studies have consistently shown that patients with breast cancer do not have equal access to treatment, with Black women being more likely to receive non–guideline-concordant therapy, lower dose intensity of chemotherapy, and lower rates of local therapy.^11–14^

Studies have described racial or socioeconomic disparities in immunotherapy receipt after approval of such treatments for patients with metastatic melanoma, non–small cell lung cancer, kidney cell carcinoma, or rectal adenocarcinoma.^15–19^ Sussman et al^15^ reported that Black patients with stage IV melanoma were more likely to have received immunotherapy than Asian or White patients. A study of node-metastatic advanced hepatocellular carcinoma found that Black patients had a 29% lower odds of immunotherapy receipt than White patients.^19^ However, current trends in immunotherapy use, presence of disparities in immunotherapy receipt, and the associated oncologic outcomes for TNBC are unknown. To close the knowledge gaps, we aimed to assess temporal trends and racial and ethnic disparities in immunotherapy use for TNBC and differences in pCR and OS among patients with TNBC receiving immunotherapy.

## Methods

### Design, Setting, and Participants

This retrospective cohort study analyzed clinical setting data from the 2017–2021 NCDB. The NCDB is a joint project of the Commission on Cancer of the American College of Surgeons and the American Cancer Society^20^ and a hospital-based cancer registry that captures approximately 72% of US new cancer diagnoses from more than 1500 Commission on Cancer–accredited programs annually.^21,22^ This study was exempt from The University of Chicago Institutional Review Board oversight with a waiver of informed consent because the NCDB contains deidentified data that do not identify hospitals, health care professionals, and patients. We followed the Strengthening the Reporting of Observational Studies in Epidemiology (STROBE) reporting guideline.^23^

The 2021 NCDB Participant User File was reviewed by 3 of us (J.Q.F., D.H., and F.M.H.). The overall inclusion criteria for this study were patients who were aged 18 years or older and diagnosed with invasive breast carcinoma between 2019 and 2021. We identified these diagnoses by reviewing the *International Classification of Diseases for Oncology* (*Third Edition*) codes, including C50.0-C50.9 (except for 9727, 9732, 9741–9742, 9749, 9762–9809, 9832, 9840–9931, 9945–9946, 9950–9968, and 9975–9993), for breast as the primary site recorded in the NCDB.^20^ According to the NCDB, the immunotherapy category consists of biological or chemical agents that change the immune system or the patient’s response to tumor cells, and this classification includes immune checkpoint inhibitors (eg, atezolizumab and pembrolizumab), *ERBB2* (formerly *HER2* or *HER2/neu*)–targeted drugs (eg, trastuzumab and pertuzumab), and antibody-drug conjugates (eg, trastuzumab deruxtecan and sacituzumab govitecan).

To more specifically identify patients treated with checkpoint inhibitors, our study focused on patients with TNBC who received concurrent immunotherapy and chemotherapy. Only atezolizumab and pembrolizumab were approved in combination with chemotherapy for TNBC during the study timeframe. Commission on Cancer–accredited cancer programs are required to report immunotherapy the patients received. Immunotherapy receipt was defined as whether it was administered as the first course of treatment or was not part of the planned first course of treatment per the NCDB. In the analysis of immunotherapy receipt and pCR for esTNBC, we included patients with stage II or III TNBC treated with NACT in 2021. In the analysis of immunotherapy receipt and OS for mTNBC, we included patients with stage IV TNBC treated with chemotherapy alone or both chemotherapy and immunotherapy from 2019 to 2021. To evaluate trends in immunotherapy use, we included the 2017–2021 data for both TNBC cohorts.

### Outcome Measures and Covariates

Rate of receipt of immunotherapy was defined as the proportion of patients receiving immunotherapy (as above) in the esTNBC and mTNBC cohorts. Pathologic CR (ypT0/Tis ypN0) was defined as the absence of invasive tumors in the breast and/or axillary lymph nodes based on the histopathologic examination.^24^ Overall survival was event or censored at the time of death from any cause or last known contact. Mortality information was not available for patients with TNBC diagnosed in 2021 because of limited follow-up time per the NCDB.

The main independent variable of interest was race and ethnicity. Per the NCDB, race and ethnicity information was self-reported. Patients reported their primary race and Spanish or Hispanic origin separately. In this analysis, we further categorized race and ethnicity into 5 groups: non-Hispanic Asian or Pacific Islander (hereinafter, Asian or Pacific Islander), non-Hispanic Black (hereinafter, Black), Hispanic, non-Hispanic White (hereinafter, White), and other. Due to a limited number of American Indian or Alaska Native patients (only 14 patients in the mTNBC cohort), this group was included in the other designation, as regression coefficients for this group could not be estimated. Other is a racial and ethnic group listed in the NCDB that represents patients who were classified as other by local cancer registries. The NCDB does not specifically define race and ethnicity classified into the other category.

Covariates included age at diagnosis, insurance status, percentage with no high school degree quartile, median household income quartile, rural-urban area, facility type, Charlson-Deyo Comorbidity Index, histologic type, and tumor grade. Per the NCDB, type of health insurance collected at the time of diagnosis and/or treatment was coded as Medicaid, Medicare, other, private or managed care, or uninsured. The percentage with no high school degree quartile (ie, ≥17.6%, 10.9%−17.5%, 6.3%−10.8%, and <6.3%) was defined as educational attainment for patients’ residential areas and measured by matching the zip code of the patients recorded at the time of diagnosis against files derived from the 2016 American Community Survey data. The median household income quartile was measured based on the 2016 American Community Survey data, spanning from 2012 to 2016 and adjusted for 2016 inflation, and was classified into less than $40 227, $40 227 to $50 353, $50 354 to $63 332, or $63 333 or greater. Rural-urban area, categorized as metropolitan, rural, and urban, was measured by matching the state and county Federal Information Processing Standards code of the patient recorded at the time of diagnosis against 2013 files published by the US Department of Agriculture Economic Research Service. Facility types were reported to the NCDB and were assigned classifications by the Commission on Cancer– accredited cancer programs: academic/research, community, comprehensive community, and integrated network. The Charlson-Deyo Comorbidity Index was categorized into groups with scores of 0, 1, and 2 or more.^25^

### Statistical Analysis

Patient characteristics are described using summary statistics. Two separate multivariable logistic regression models were fit to examine racial and ethnic differences in immunotherapy receipt for each TNBC cohort. A stepwise regression approach was implemented. Model 1 included race and ethnicity, age at diagnosis, histologic type, tumor grade, clinical T category, and clinical nodal status. Model 2 included Charlson-Deyo Comorbidity Index, percentage with no high school degree quartile, median household income quartile, rural-urban area, type of health insurance, and facility type, in addition to the variables contained in model 1. To evaluate the association between immunotherapy use and pCR, we conducted logistic regression, controlling for age at diagnosis, race and ethnicity, histologic type, and tumor grade. Adjusted odds ratios (AORs) and 95% CIs were calculated. Regarding survival analysis, we used the Kaplan-Meier method to calculate the median survival time (in months) and compared Kaplan-Meier curves using the log-rank test. To examine racial and ethnic differences in OS, 2 Cox proportional hazards regression models were fit using a similar stepwise approach. For model 1, we included age at diagnosis, race and ethnicity, histologic type, tumor grade, and Charlson-Deyo Comorbidity Index. Model 2 was additionally controlled for percentage with no high school degree quartile, median household income quartile, type of health insurance, and facility type. Adjusted hazard ratios (AHRs) and 95% CIs were computed. A 2-sided value of *P* < .05 was considered statistically significant. All analyses were performed using Stata, version 18.0 (StataCorp LLC). Data were analyzed from April 1 to August 31, 2024.

## Results

### Patient Characteristics

The overall sample characteristics of patients with TNBC are described in eTable 1 in Supplement 1.A total of 10 724 patients from the 2019–2021 NCDB were included, with a mean (SD) age of 56.1 (13.7) years. Overall, 473 patients (4.4%) were Asian or Pacific Islander, 2569 (24.0%) were Black, 981 (9.1%) were Hispanic, 6465 (60.3%) were White, and 236 (2.2%) were other. A total of 1416 individuals (13.2%) were covered by Medicaid, 3155 (29.4%) were covered by Medicare, 5588 (52.1%) had private insurance, and 330 (3.1%) were uninsured. Classification of the cancer programs was 3201 (34.6%) academic/research, 595 (6.4%) community, 3382 (36.6%) comprehensive community, and 2065 (22.3%) integrated network (eTable 1 in Supplement 1).

### Trends in Immunotherapy Use

Overall, immunotherapy use increased from 5.5% in 2017 to 38.8% in 2021 for mTNBC and from 4.2% in 2017 to 48.0% in 2021 for esTNBC (Figure). This finding is consistent with the timeframe of approval in both TNBC settings and rates of PD-L1 positivity for mTNBC. Similar increasing patterns were observed across racial and ethnic groups; however, the increases in both TNBC cohorts were relatively lower among Black patients during the same period (eTable 2 in Supplement 1).

### Immunotherapy and pCR in esTNBC

We identified 7655 patients with esTNBC in 2021 (mean [SD] age, 54.5 [13.5] years). Detailed characteristics are presented in eTable 1 in Supplement 1. Overall, 3662 patients (48.0%) received immunotherapy; of these, 201 of 376 (53.5%) Asian or Pacific Islander patients, 788 of 1715 (45.9%) Black patients, 358 of 738 (48.5%) Hispanic patients, 2228 of 4625 (48.2%) White patients, and 87 of 180 (48.3%) patients with other race received immunotherapy (eTable 3 in Supplement 1). Asian or Pacific Islander, Black, or Hispanic patients experienced longer mean and median numbers of days between diagnosis and immunotherapy initiation than White patients. After adjustment for age, histologic subtype, and grade (Table 1; model 1), Black patients had lower odds of immunotherapy receipt than White patients (AOR, 0.88; 95% CI, 0.78–0.99); however, the difference was not significant when further controlling for socioeconomic factors (AOR, 0.95; 95% CI, 0.82–1.11) (Table 1; model 2). Compared with White patients, Asian or Pacific Islander (AOR, 1.04; 95% CI, 0.78–1.38), Hispanic (AOR, 0.97; 95% CI, 0.77–1.22), and patients with other race (AOR, 0.89; 95% CI, 0.61–1.30) had similar odds of having received immunotherapy. Patients without insurance had lower odds of immunotherapy receipt than those privately insured (AOR, 0.62; 95% CI, 0.40–0.96). Comprehensive community programs had lower odds of immunotherapy use compared with academic/research programs (AOR, 0.81; 95% CI, 0.70–0.93) (Table 1; model 2).

Patients treated with both NACT and immunotherapy achieved a greater pCR rate than those treated with NACT alone (AOR, 1.45; 95% CI, 1.31–1.61) (eTable 4 in Supplement 1). Among patients receiving immunotherapy, the odds of pCR were not significantly different between Asian or Pacific Islander and White patients (AOR, 0.82; 95% CI, 0.53–1.26), between Black and White patients (AOR, 0.92; 95% CI, 0.73–1.17), between Hispanic and White patients (AOR, 1.27; 95% CI, 0.90–1.79), or between other and White patients (AOR, 1.09; 95% CI, 0.60–1.96) (eTable 5 in Supplement 1).

### Immunotherapy and OS in mTNBC

We identified 3069 patients with mTNBC from 2019 to 2021 (mean [SD] age, 59.8 [13.7] years). eTable 1 in Supplement 1 provides detailed characteristics. Among 1021 patients (33.3%) who received immunotherapy, 36 of 97 (37.1%) Asian or Pacific Islander patients, 238 of 848 (28.1%) Black patients, 81 of 241 (33.6%) Hispanic patients, 649 of 1828 (35.5%) White patients, and 17 of 55 (30.9%) patients with other race received immunotherapy (eTable 3 in Supplement 1). Compared with White patients, Black patients had a longer median number of days between diagnosis and immunotherapy initiation, while Asian or Pacific Islander and Hispanic patients had shorter mean and median numbers of days. After covariate adjustment, Black patients remained at lower odds of immunotherapy receipt than White patients (AOR, 0.63; 95% CI, 0.49–0.80) (Table 2; model 2). The odds of immunotherapy receipt were not significantly different between Asian or Pacific Islander and White patients (AOR, 1.33; 95% CI, 0.78–2.27), between Hispanic and White patients (AOR, 0.84; 95% CI, 0.55–1.28), or between White patients and patients with other race (AOR, 0.70; 95% CI, 0.32–1.55) (Table 2; model 2). Comprehensive community programs had lower odds of immunotherapy use compared with academic/research programs (AOR, 0.79; 95% CI, 0.63–0.99).

With a median follow-up of 14.8 (IQR, 7.5–26.0) months, patients treated with both chemotherapy and immunotherapy had a longer median survival time (eFigure, eTable 6 in Supplement 1) and a lower mortality risk (AHR, 0.80; 95% CI, 0.68–0.94) than those treated with chemotherapy alone (eTable 7 in Supplement 1). Among patients receiving immunotherapy, OS was not significantly different between Asian or Pacific Islander and White patients (AHR, 0.50; 95% CI, 0.20–1.26), between Black and White patients (AHR, 0.92; 95% CI, 0.64–1.32), between Hispanic and White patients (AHR, 0.48; 95% CI, 0.23–1.00), or between White patients and patients with other race (AHR, 2.04; 95% CI, 0.85–4.87) (Table 3; model 2), although the sample size was small for groups aside from Black and White patients, leading to insufficient power to evaluate all but the largest mortality differences.

## Discussion

To our knowledge, this is the first study to examine trends and racial and ethnic disparities in immunotherapy use for TNBC and racial and ethnic differences in pCR and OS among patients with TNBC receiving immunotherapy. Significant increases in immunotherapy use from 2017 to 2021 were observed in esTNBC and mTNBC. The early uptake of immunotherapy in 2017 and 2018 may be due to published data before the US Food and Drug Administration formally approved checkpoint inhibitors for triple-negative breast cancer. Positive results from the IMpassion130 trial were first announced and published in 2018.^26^ It is also possible that some patients from the NCDB were enrolled in immunotherapy clinical trials. There may also have been a low rate of patients who received non–checkpoint blockade therapies, coded as immunotherapy by the NCDB (eg, bevacizumab or trastuzumab). The lack of annotation of specific immunotherapy agents is a limitation of this study. Nonetheless, the rapid increase in immunotherapy use for TNBC coincides with the expanded approval of PD-1/PD-L1 inhibitors based on the KEYNOTE-355, KEYNOTE-522, and IMpassion130 trials.^7,9,26^ The rate of immunotherapy use for mTNBC is overall consistent with rates of PD-L1 positivity (reported as 38% in KEYNOTE-355).^7^ The rate of immunotherapy use for esTNBC is also as expected given the accelerated approval of pembrolizumab that occurred in mid-2021, and we would expect this rate to increase to closer to 100% in 2024.^27^

We found that Black patients with mTNBC were less likely to have received immunotherapy than White patients, consistent with previous research on racial disparities in receipt of surgery or chemotherapy for TNBC.^28^ Moreover, we observed that Black patients in both TNBC cohorts, as well as Asian or Pacific Islander and Hispanic patients with esTNBC, experienced a longer time between diagnosis and immunotherapy initiation compared with White patients. A study reported that Black patients with stage IV non–small cell lung cancer were 12% less likely than White patients to initiate immunotherapy within a month at the end of life, and this disparity varied across facility types and volumes.^16^ Similarly, these racial and ethnic disparities in immunotherapy receipt for esTNBC in our study were largely due to socioeconomic factors, such as type of health insurance and facility type, as no substantial racial and ethnic differences were seen after controlling for these factors. Oncology programs should consider addressing these barriers to timely immunotherapy initiation for TNBC across racial and ethnic groups.

However, in the mTNBC setting, Black patients were approximately 37% less likely than White patients to have received immunotherapy, even after controlling for both clinicopathologic and socioeconomic characteristics. There are other unmeasured factors that could be associated with this persistent difference in immunotherapy use, such as racial differences in PD-L1 status, differences in the assays used to determine PD-L1 status, or differences in access to biomarker testing. A study of TNBC tissue samples found significantly greater PD-L1 percent positivity in patients with African ancestry than in those without.^29^ In contrast, the IMpassion130 trial reported a slightly lower rate of PD-L1–positive tumors in Black patients than in White patients (38.9% vs 41.7%).^30^ Another study analyzing a small sample of TNBC tissues revealed that patients with African ancestry (42.4%) had lower PD-L1 expression than those with European (51.6%) or Central/South American ancestry (46.7%), although the difference was not statistically significant.^31^ Regional differences in choice of PD-L1 assay could affect differences in immunotherapy use. The VENTANA SP142 assay was an approved companion diagnostic for atezolizumab and the DAKO 22C3 PD-L1 assay was approved for pembrolizumab during the study timeframe, and these assays have different rates of positive results.^32^ Preexisting autoimmune disease may also play a role in decisions to forgo immunotherapy, given the higher prevalence of autoimmune conditions, such as multiple sclerosis and lupus, in Black women.^33^ Future studies are needed to quantify systemic barriers, such as treatment costs, lack of access to biomarker testing, and other social determinants stemming from systemic racism, which may influence immunotherapy use.^34^

In the esTNBC cohort, patients treated with both NACT and immunotherapy achieved a higher pCR rate than those treated with NACT alone, which is aligned with results from the KEYNOTE-522 and IMpassion130 trials.^9,26^ An important finding of this study is that Black patients with esTNBC receiving immunotherapy achieved a similar pCR rate as White patients, in contrast to the 15% decrease in pCR rates among Black patients with esTNBC treated with NACT alone in a similarly adjusted analysis.^4^ In the mTNBC cohort, we observed that patients treated with chemotherapy and immunotherapy had better OS than those treated with chemotherapy alone, congruent with randomized clinical trial data and clinical setting analyses.^7,15,35,36^ We also found that among patients receiving immunotherapy, Black patients had a similar risk of mortality as White patients. Collectively, these findings confirm the benefits of immunotherapy for TNBC and that equal access to immunotherapy might help mitigate racial disparities in treatment outcomes.

### Limitations

This study has several limitations, particularly regarding its retrospective design and unmeasured confounders. The specific immunotherapy agent administered is not listed in the NCDB, and this immunotherapy category in the registry includes other biological agents, such as bevacizumab, *ERBB2*–targeted therapies, and sacituzumab govitecan. However, as our study focused on TNBC receiving concurrent chemotherapy, these therapies likely represent a minimal fraction of patients receiving immunotherapy. The current study is also limited by the lack of PD-L1 expression data, and it is unclear whether the racial differences in immunotherapy receipt for mTNBC are due to local practice patterns or differences in PD-L1 expression between racial groups.^29–31^ Therefore, this warrants future research on the intersection of these key unmeasured factors. In addition, the number of patients receiving immunotherapy was low, and thus, this study does not rule out small racial and ethnic differences in pCR for esTNBC or OS for mTNBC among patients receiving immunotherapy.

## Conclusions

In this cohort study of TNBC, our findings highlighted increasing trends and racial and ethnic disparities in immunotherapy use for TNBC. Immunotherapy receipt for esTNBC was associated in part with facility type and insurance status. For mTNBC, Black patients were less likely to have received immunotherapy, which could imply racial differences in PD-L1 expression, although reasons for these disparities are needed in future research. Receiving immunotherapy could improve pCR in esTNBC and OS in mTNBC. Our study provides insights into potential strategies to ameliorate equitable access to immunotherapy to help mitigate racial and ethnic disparities in oncologic outcomes for TNBC.

## Supplementary Material

Supplemental Online Content **eTable 1.** Overall Characteristics of Triple-Negative Breast Cancer in the 2019–2021 National Cancer Database
**eTable 2.** Estimated Prevalence of Immunotherapy Use Among Patients With Triple-Negative Breast Cancer in the 2017–2021 National Cancer Database, by Stage and Year of Diagnosis, Overall, and by Race and Ethnicity
**eTable 3.** Estimated Prevalence of Immunotherapy Use Among Patients With Triple-Negative Breast Cancer, by Stage and Race/Ethnicity
**eTable 4.** Association Between Immunotherapy Use and Pathologic Complete Response Among Patients With Stage II-III, Triple-Negative Breast Cancer in the 2021 National Cancer Database
**eTable 5.** Assessment of Racial and Ethnic Differences in Pathologic Complete Response Among Patients With Stage II-III, Triple-Negative Breast Cancer Who Received Immunotherapy and Neoadjuvant Chemotherapy in the 2021 National Cancer Database
**eTable 6.** Kaplan-Meier Estimate of Median Overall Survival Time Among Patients With Stage IV, Triple-Negative Breast Cancer With Chemotherapy in the 2019–2021 National Cancer Database
**eTable 7.** Immunotherapy Use and Overall Survival Among Patients With Stage IV, Triple-Negative Breast Cancer With Chemotherapy in the 2019–2021 National Cancer Database

Supplemental Online Content 2

## Figures and Tables

**Figure.**
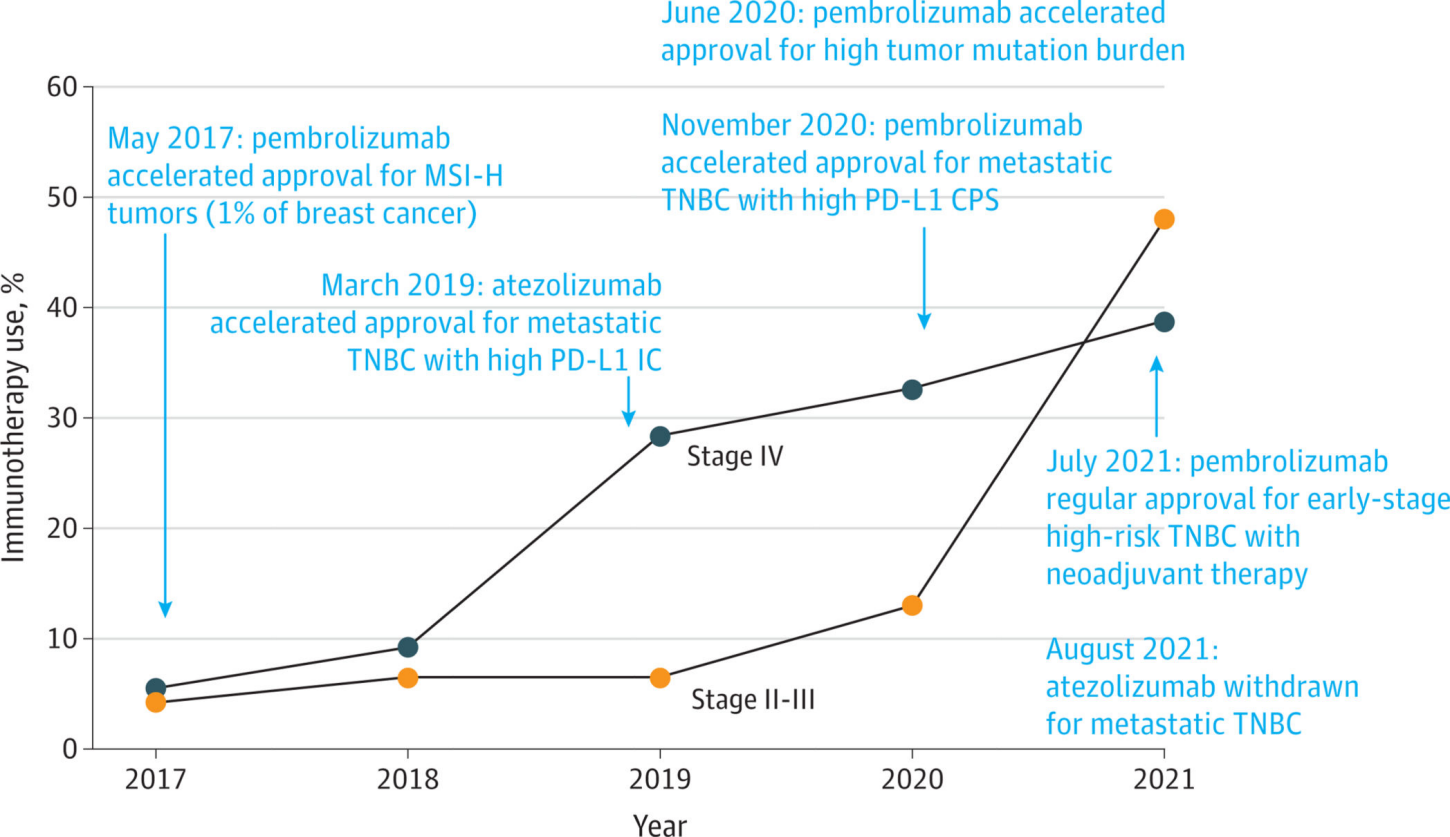
Estimated Prevalence of Immunotherapy Use Among Patients With Triple-Negative Breast Cancer by Stage CPS indicates combined positive score; IC, intracellular score; MSI-H, microsatellite instability-high; PD-L1, programmed death-ligand 1; TNBC, triple-negative breast cancer.

**Table 1.** Characteristics Associated With Immunotherapy Use Among Patients With Stage II to III Triple-Negative Breast Cancer in the 2021 NCDB

	Logistic regression			
	Model 1		Model 2	
Characteristic	AOR (95% CI)^a^	*P* value	AOR (95% CI)^b^	*P* value

Race and ethnicity				

Asian or Pacific Islander	1.15 (0.92–1.43)	.22	1.04 (0.78–1.38)	.79

Black	0.88 (0.78–0.99)	.03	0.95 (0.82–1.11)	.55

Hispanic	0.89 (0.75–1.05)	.16	0.97 (0.77–1.22)	.80

White	1 [Reference]	NA	1 [Reference]	NA

Other^c^	0.90 (0.66–1.23)	.52	0.89 (0.61–1.30)	.54

Age at diagnosis, per 10-y increase	0.83 (0.80–0.86)	<.001	0.86 (0.80–0.93)	<.001

Histologic type				

Ductal	1 [Reference]	NA	1 [Reference]	NA

Ductal and lobular	1.15 (0.67–1.99)	.62	1.38 (0.68–2.79)	.37

Inflammatory breast cancer	1.28 (0.89–1.86)	.18	1.15 (0.74–1.80)	.53

Lobular	1.04 (0.63–1.71)	.87	1.25 (0.70–2.25)	.45

Metaplastic breast cancer	1.00 (0.72–1.39)	.99	0.93 (0.63–1.38)	.74

Other	1.12 (0.72–1.74)	.61	1.16 (0.68–1.99)	.59

Tumor grade				

1	1 [Reference]	NA	1 [Reference]	NA

2	1.35 (0.71–2.58)	.37	1.39 (0.63–3.08)	.42

3	1.37 (0.72–2.59)	.34	1.37 (0.62–3.00)	.43

AJCC clinical T category				

cT2	1 [Reference]	NA	1 [Reference]	NA

cT3	1.25 (1.09–1.43)	.001	1.20 (1.01–1.42)	.04

cT4	1.10 (0.88–1.38)	.40	1.20 (0.91–1.58)	.19

AJCC clinical nodal status				

Negative (cN0)	1 [Reference]	NA	1 [Reference]	NA

Positive (cN1+)	1.33 (1.20–1.47)	<.001	1.33 (1.18–1.51)	<.001

Type of health insurance				

Medicaid	NA	NA	0.96 (0.78–1.17)	.68

Medicare	NA	NA	0.86 (0.72–1.03)	.09

Other government/unknown	NA	NA	0.87 (0.57–1.31)	.50

Private/managed care	NA	NA	1 [Reference]	NA

Uninsured	NA	NA	0.62 (0.40–0.96)	.03

Type of cancer program				

Academic/research	NA	NA	1 [Reference]	NA

Community	NA	NA	1.18 (0.91–1.53)	.22

Comprehensive community	NA	NA	0.81 (0.70–0.93)	.003

Integrated network	NA	NA	1.00 (0.85–1.18)	.98

Abbreviations: AJCC, American Joint Committee on Cancer; AOR, adjusted odds ratio; NA, not applicable; NCDB, National Cancer Database.

a Model 1 included race and ethnicity, age at diagnosis, histologic type, and tumor grade.

b Model 2 included race and ethnicity, age at diagnosis, histologic type, tumor grade, the Charlson-Deyo Comorbidity Index, percentage with no high school degree quartile, median household income quartile, type of health insurance, rural-urban area, and facility type.

c Other is a racial and ethnic group listed in the NCDB that represents patients who were classified as other by local cancer registries. The NCDB does not specifically define race and ethnicity classified as other.

**Table 2.** Characteristics Associated With Immunotherapy Use Among Patients With Stage IV Triple-Negative Breast Cancer in the 2019–2021 NCDB

	Logistic regression			
	Model 1		Model 2	
Characteristic	AOR (95% CI)^a^	*P* value	AOR (95% CI)^b^	*P* value

Race and ethnicity				

Asian or Pacific Islander	1.22 (0.76–1.95)	.42	1.33 (0.78–2.27)	.30

Black	0.69 (0.57–0.84)	<.001	0.63 (0.49–0.80)	<.001

Hispanic	0.82 (0.60–1.13)	.23	0.84 (0.55–1.28)	.41

White	1 [Reference]	NA	1 [Reference]	NA

Other^c^	0.97 (0.52–1.81)	.92	0.70 (0.32–1.55)	.38

Age at diagnosis, per 10-y increase	0.93 (0.88–0.99)	.03	0.96 (0.86–1.07)	.45

Histologic type				

Ductal	1 [Reference]	NA	1 [Reference]	NA

Ductal and lobular	1.03 (0.51–2.10)	.93	1.68 (0.76–3.71)	.20

Inflammatory breast cancer	1.17 (0.92–1.50)	.21	1.03 (0.77–1.39)	.83

Lobular	0.68 (0.39–1.18)	.17	0.63 (0.33–1.22)	.17

Metaplastic breast cancer	0.88 (0.49–1.57)	.66	0.59 (0.27–1.28)	.18

Other	0.90 (0.57–1.41)	.64	0.90 (0.54–1.50)	.68

Tumor grade				

1	1 [Reference]	NA	1 [Reference]	NA

2	1.52 (0.59–4.71)	.47	0.89 (0.26–3.06)	.86

3	1.69 (0.55–5.21)	.36	0.99 (0.29–3.35)	.99

Type of health insurance				

Medicaid	NA	NA	0.88 (0.65–1.19)	.41

Medicare	NA	NA	0.92 (0.69–1.23)	.59

Other government/unknown	NA	NA	0.82 (0.42–1.62)	.57

Private/managed care	NA	NA	1 [Reference]	NA

Uninsured	NA	NA	0.78 (0.48–1.28)	.33

Type of cancer program				

Academic/research	NA	NA	1 [Reference]	NA

Community	NA	NA	0.72 (0.47–1.09)	.12

Comprehensive community	NA	NA	0.79 (0.63–0.99)	.04

Integrated network	NA	NA	1.20 (0.92–1.57)	.18

Abbreviations: AOR, adjusted odds ratio; NA, not applicable; NCDB, National Cancer Database.

a Model 1 included race and ethnicity, age at diagnosis, histologic type, and tumor grade.

b Model 2 included race and ethnicity, age at diagnosis, histologic type, tumor grade, the Charlson-Deyo Comorbidity Index, percentage with no high school degree quartile, median household income quartile, type of health insurance, rural-urban area, and facility type.

c Other is a racial and ethnic group listed in the NCDB that represents patients who were classified as other by local cancer registries. The NCDB does not specifically define race and ethnicity classified as other.

**Table 3.** Racial and Ethnic Differences in Overall Survival Among Patients With Stage IV Triple-Negative Breast Cancer Who Received Immunotherapy in the 2019–2021 NCDB

	Cox proportional hazards regression			
	Model 1		Model 2	
Characteristic	AOR (95% CI)^a^	*P* value	AOR (95% CI)^b^	*P* value

Age at diagnosis, per 10-y increase	1.07 (0.98–1.18)	.12	1.14 (0.97–1.35)	.11

Race and ethnicity				

Asian or Pacific Islander	0.53 (0.26–1.09)	.08	0.50 (0.20–1.26)	.14

Black	0.93 (0.71–1.24)	.63	0.92 (0.64–1.32)	.63

Hispanic	0.67 (0.40–1.13)	.13	0.48 (0.23–1.00)	.05

White	1 [Reference]	NA	1 [Reference]	NA

Other^c^	0.82 (0.36–1.87)	.64	2.04 (0.85–4.87)	.11

Histologic type				

Ductal	1 [Reference]	NA	1 [Reference]	NA

Ductal and lobular	1.21 (0.50–2.95)	.68	1.43 (0.57–3.64)	.45

Inflammatory breast cancer	1.52 (1.09–2.11)	.01	1.41 (0.93–2.12)	.10

Lobular	1.70 (0.85–3.42)	.13	1.61 (0.65–3.95)	.30

Metaplastic breast cancer	0.65 (0.26–1.58)	.34	0.95 (0.34–2.63)	.92

Other	1.35 (0.75–2.43)	.32	1.31 (0.65–2.64)	.46

Tumor grade				

1	1 [Reference]	NA	1 [Reference]	NA

2	1.26 (0.17–9.40)	.82	1.24 (0.16–9.56)	.84

3	1.33 (0.18–9.71)	.78	1.45 (0.19–11.00)	.72

Charlson-Deyo Comorbidity Index				

0	1 [Reference]	NA	1 [Reference]	NA

1	1.10 (0.79–1.53)	.56	1.06 (0.72–1.56)	.77

≥2	1.33 (0.74–2.40)	.34	0.96 (0.48–1.92)	.90

Type of health insurance				

Medicaid	NA	NA	1.32 (0.85–2.07)	.22

Medicare	NA	NA	1.04 (0.69–1.57)	.84

Other government/unknown	NA	NA	0.52 (0.17–1.54)	.24

Private/managed care	NA	NA	1 [Reference]	NA

Uninsured	NA	NA	1.93 (0.95–3.91)	.07

Type of cancer program				

Academic/research	NA	NA	1 [Reference]	NA

Community	NA	NA	1.69 (0.93–3.07)	.09

Comprehensive community	NA	NA	1.62 (1.17–2.25)	.004

Integrated network	NA	NA	1.28 (0.88–1.87)	.19

Abbreviations: AHR, adjusted hazard ratio; NA, not applicable; NCDB, National Cancer Database.

a Model 1 included race and ethnicity, age at diagnosis, histologic type, tumor grade, and the Charlson-Deyo Comorbidity Index.

b Model 2 included race and ethnicity, age at diagnosis, histologic type, tumor grade, the Charlson-Deyo Comorbidity Index, percentage with no high school degree quartile, median household income quartile, type of health insurance, and facility type.

c Other is a racial and ethnic group listed in the NCDB that represents patients who were classified as other by local cancer registries. The NCDB does not specifically define race and ethnicity classified as other.

## Data Availability

See Supplement 2.

## Key Points

### Question

Are there racial and ethnic differences in the use of immunotherapy for triple-negative breast cancer (TNBC)?

### Findings

In a cohort study that included 7655 patients with early-stage TNBC in 2021 and 3069 patients with metastatic TNBC from 2019 to 2021, rates of immunotherapy by race and ethnicity were similar in early-stage TNBC, but lower among Black patients with metastatic TNBC. The overall survival rate between Black and White patients receiving immunotherapy was similar.

### Meaning

Although there were racial differences in the use of immunotherapy, Black patients receiving immunotherapy attained similar outcomes to White patients; these findings suggest that efforts should be made to ensure equal access to immunotherapy.
