# Supplementary material for: Trends and Disparities in the Use of Immunotherapy for Triple-Negative Breast Cancer in the US
Source: JAMA Netw Open. Author manuscript; Available in PMC 2025 Mar 25. (PMC11833518; doi:10.1001/jamanetworkopen.2024.60243)
Supplement: Supplemental Online Content — eTable 1. Overall Characteristics of Triple-Negative Breast Cancer in the 2019–2021 National Cancer Database eTable 2. Estimated Prevalence of Immunotherapy Use Among Patients With Triple-Negative Breast Cancer in the 2017–2021 National Cancer Database, by Stage and Year of Diagnosis, Overall, and by Race and Ethnicity eTable 3. Estimated Prevalence of Immunotherapy Use Among Patients With Triple-Negative Breast Cancer, by Stage and Race/Ethnicity eTable 4. Association Between Immunotherapy Use and Pathologic Complete Response Among Patients With Stage II-III, Triple-Negative Breast Cancer in the 2021 National Cancer Database eTable 5. Assessment of Racial and Ethnic Differences in Pathologic Complete Response Among Patients With Stage II-III, Triple-Negative Breast Cancer Who Received Immunotherapy and Neoadjuvant Chemotherapy in the 2021 National Cancer Database eTable 6. Kaplan-Meier Estimate of Median Overall Survival Time Among Patients With Stage IV, Triple-Negative Breast Cancer With Chemotherapy in the 2019–2021 National Cancer Database eTable 7. Immunotherapy Use and Overall Survival Among Patients With Stage IV, Triple-Negative Breast Cancer With Chemotherapy in the 2019–2021 National Cancer Database [file NIHMS2058962-supplement-Supplemental_Online_Content.pdf]

Supplemental Online Content

eTable 1. Overall Characteristics of Triple-Negative Breast Cancer in the 2019-2021 National Cancer Database

eTable 2. Estimated Prevalence of Immunotherapy Use among Patients With Triple-Negative Breast Cancer in the 2017-2021 National Cancer Database, by Stage and Year of Diagnosis, Overall and by Race and Ethnicity

eTable 3. Estimated Prevalence of Immunotherapy Use among Patients With Triple-Negative Breast Cancer, by Stage and Race/Ethnicity

eTable 4. Association between Immunotherapy Use and Pathologic Complete Response among Patients With Stage II-III, Triple-Negative Breast Cancer in the 2021 National Cancer Database

eTable 5. Assessment of Racial and Ethnic Differences in Pathologic Complete Response among Patients With Stage II-III, Triple-Negative Breast Cancer Who Received Immunotherapy and Neoadjuvant Chemotherapy in the 2021 National Cancer Database

eFigure 1. Kaplan-Meier Curves of Overall Survival among Patients With Stage IV, Triple-Negative Breast Cancer with Chemotherapy, by Immunotherapy Use in the 2019-2021 National Cancer Database

eTable 6. Kaplan-Meier Estimate of Median Overall Survival Time among Patients With Stage IV, Triple-Negative Breast Cancer with Chemotherapy in the 2019-2021 National Cancer Database

eTable 7. Immunotherapy Use and Overall Survival among Patients With Stage IV, Triple-Negative Breast Cancer with Chemotherapy in the 2019-2021 National Cancer Database

eTable 1. Overall Characteristics of Triple-Negative Breast Cancer in the 2019-2021 National Cancer Database

	**National Cancer Database, 2019-2021**	**National Cancer Database, 2021**	**Overall**
	**Stage IV**	**Stage II-III**	**Stage II, III and IV**
**Characteristic**	(n=3069)	(n=7655)	(N=10724)
	No. (%)	No. (%)	No. (%)
**Age at diagnosis**			
Mean (SD)	59.8 (13.7)	54.5 (13.5)	56.1 (13.7)
Median (IQR)	60.0 (51.0, 70.0)	55.0 (44.0, 65.0)	56.0 (46.0, 66.0)
**Race/Ethnicity**			
Asian or Pacific Islander	97 (3.2)	376 (4.9)	473 (4.4)
Black	848 (27.6)	1721 (22.5)	2569 (24.0)
Hispanic	241 (7.9)	740 (9.7)	981 (9.1)
White	1828 (59.6)	4637 (60.6)	6465 (60.3)
Other	55 (1.8)	181 (2.4)	236 (2.2)
**Histologic type**			
Ductal	2279 (74.3)	7041 (92.0)	9320 (86.9)
Ductal and lobular	36 (1.2)	61 (0.8)	97 (0.9)
Inflammatory breast cancer	362 (11.8)	198 (2.6)	560 (5.2)
Lobular	90 (2.9)	75 (1.0)	165 (1.5)
Metaplastic breast cancer	72 (2.3)	177 (2.3)	249 (2.3)
Other	230 (7.5)	103 (1.3)	333 (3.1)
**Tumor grade**			
1	18 (0.7)	47 (0.6)	65 (0.6)
2	459 (17.7)	1127 (15.0)	1586 (15.7)
3	2113 (81.6)	6349 (84.4)	8462 (83.7)
**Charlson-Deyo Comorbidity Index**			
0	2427 (79.1)	6320 (82.6)	8747 (81.6)
1	442 (14.4)	964 (12.6)	1406 (13.1)
≥2	200 (6.5)	371 (4.8)	571 (5.3)
**Percent no high school degree quartiles [a]**			

≥17.6%	651 (25.2)	1291 (20.6)	1942 (22.0)
10.9%-17.5%	737 (28.5)	1588 (25.4)	2325 (26.3)
6.3%-10.8%	660 (25.5)	1791 (28.6)	2451 (27.7)
<6.3%	538 (20.8)	1582 (25.3)	2120 (24.0)
**Median household income quartiles [b]**			
<$40,227	617 (23.9)	1103 (17.7)	1720 (19.5)
$40,227–$50,353	582 (22.6)	1284 (20.6)	1866 (21.1)
$50,354–$63,332	543 (21.0)	1421 (22.8)	1964 (22.3)
≥$63,333	838 (32.5)	2435 (39.0)	3273 (37.1)
**Type of health insurance**			
Medicaid	475 (15.5)	941 (12.3)	1416 (13.2)
Medicare	1160 (37.8)	1995 (26.1)	3155 (29.4)
Other government/unknown	69 (2.2)	166 (2.2)	235 (2.2)
Private/managed care	1212 (39.5)	4376 (57.2)	5588 (52.1)
Uninsured	153 (5.0)	177 (2.3)	330 (3.1)
**Rural/urban area [c]**			
Metropolitan	2616 (86.7)	6548 (87.4)	9164 (87.2)
Rural	45 (1.5)	89 (1.2)	134 (1.3)
Urban	357 (11.8)	853 (11.4)	1210 (11.5)
**Type of cancer program**			
Academic/research	1023 (36.3)	2178 (33.9)	3201 (34.6)
Community	209 (7.4)	386 (6.0)	595 (6.4)
Comprehensive community	1033 (36.7)	2349 (36.5)	3382 (36.6)
Integrated network	550 (19.5)	1515 (23.6)	2065 (22.3)

Abbreviations: SD, standard deviation; IQR, interquartile range; No., number.

Note: Other is a racial/ethnic group listed in the NCDB, which represents patients who were classified as Other by local cancer registries. The NCDB does not specifically define race/ethnicity classified into Other.

[a] Defined as education attainment for patient residence areas and measured by matching the zip code of the patient recorded at the time of diagnosis against files derived from the 2016 American Community Survey data.

[b] Based on the 2016 American Community Survey data, spanning years 2012–2016 and adjusted for 2016 inflation.

[c] Measured by matching the state and county FIPS code of the patient recorded at the time of diagnosis against 2013 files published by the United States Department of Agriculture Economic Research Service.

eTable 2. Estimated Prevalence of Immunotherapy Use among Patients With Triple-Negative Breast Cancer in the 2017-2021 National Cancer Database, by Stage and Year of Diagnosis, Overall and by Race and Ethnicity

		**Overall**	**Asian or Pacific Islander**	**Black**	**Hispanic**	**White**	**Other**
**Characteristic**		**Yes**	**Yes**	**Yes**	**Yes**	**Yes**	**Yes**
		No. (%)	No. (%)	No. (%)	No. (%)	No. (%)	No. (%)
**Stage IV**							
	**Year of diagnosis**						
	2017	54 (5.5)	1 (3.7))	12 (4.6)	4 (5.9)	36 (6.0)	1 (5.0)
	2018	91 (9.2)	5 (17.2)	18 (6.8)	7 (9.3)	59 (9.7)	2 (15.4)
	2019	291 (28.4)	5 (23.8)	67 (22.2)	16 (24.6)	194 (31.4)	9 (42.9)
	2020	337 (32.7)	18 (42.9)	80 (28.5)	27 (31.0)	210 (35.0)	2 (9.5)
	2021	393 (38.8)	13 (38.2)	91 (34.3)	38 (42.7)	245 (40.1)	6 (46.2)
**Stage II-III**							
	**Year of diagnosis**						
	2017	211 (4.2)	11 (5.5)	41 (3.7)	20 (4.4)	137 (4.3)	2 (2.5)
	2018	323 (6.5)	23 (12.2)	51 (4.6)	31 (6.4)	211 (6.8)	7 (8.2)
	2019	345 (6.5)	26 (11.2)	48 (4.1)	33 (6.4)	233 (7.0)	5 (5.4)
	2020	700 (13.0)	40 (16.3)	106 (9.2)	68 (13.2)	473 (14.0)	13 (11.4)
	2021	3662 (48.0)	201 (53.5)	788 (46.0)	358 (48.5)	2228 (48.2)	87 (48.3)

Abbreviation: No., number.
Note: Other is a racial/ethnic group listed in the NCDB, which represents patients who were classified as Other by local cancer registries. The NCDB does not specifically define race/ethnicity classified into Other.

eTable 3. Estimated Prevalence of Immunotherapy Use among Patients With Triple-Negative Breast Cancer, by Stage and Race/Ethnicity

		Receipt of immunotherapy			No. of days between initial diagnosis and start of immunotherapy			
**Characteristic**		**No (chemotherapy alone)**	**Yes (chemotherapy and immunotherapy)**					
		No. (row %)	No. (row %)	*P* value [a]	Mean (SD)	*P* value [a]	Median (IQR)	*P* value [a]
**National Cancer Database, 2019-2021**								
**Stage IV**	**Overall**	2048 (66.7)	1021 (33.3)		54.6 (39.0)		46.0 (33.0, 67.0)	
(n=3069)	**Race/Ethnicity**							
	Asian or Pacific Islander	61 (62.9)	36 (37.1)	.004	44.0 (18.9)	<.001	41.5 (30.0, 56.5)	.002
	Black	610 (71.9)	238 (28.1)		60.4 (34.0)		51.0 (37.0, 77.0)	
	Hispanic	160 (66.4)	81 (33.6)		51.6 (30.0)		44.0 (33.0, 58.0)	
	White	1179 (64.5)	649 (35.5)		52.5 (33.1)		46.0 (31.0, 63.0)	
	Other	38 (69.1)	17 (30.9)		91.6 (165.7)		52.0 (35.0, 60.0)	
**National Cancer Database, 2021**								
**Stage II-III**	**Overall**	3972 (52.0)	3662 (48.0)		67.7 (69.4)		42.0 (29.0, 74.0)	
(n=7655)	**Race/Ethnicity**							
	Asian or Pacific Islander	175 (46.5)	201 (53.5)	.11	72.5 (69.6)	<.001	45.0 (29.0-86.5)	<.001
	Black	927 (54.1)	788 (45.9)		77.4 (79.1)		48.0 (33.0-84.0)	
	Hispanic	380 (51.5)	358 (48.5)		76.3 (71.9)		49.5 (33.0-92.0)	
	White	2397 (51.8)	2228 (48.2)		62.8 (65.4)		40.0 (28.0-65.0)	
	Other	93 (51.7)	87 (48.3)		58.1 (50.2)		43.0 (25.0-76.0)	

Abbreviations: No., number; SD, standard deviation; IQR, interquartile range; API.
Note: Other is a racial/ethnic group listed in the NCDB, which represents patients who were classified as Other by local cancer registries. The NCDB does not specifically define race/ethnicity classified into Other.
[a] *P* values were calculated using ANOVA, Kruskal-Wallis, or Pearson's X^2 tests, as appropriate.

eTable 4. Association between Immunotherapy Use and Pathologic Complete Response among Patients With Stage II-III, Triple-Negative Breast Cancer in the 2021 National Cancer Database

	**Pathologic complete response**			**Logistic regression**	
	**Did not achieve**	**Achieved**			
**Characteristic**	(n=4749 [62.0%])	(n=2906 [38.0%])			
	**No. (row %)**	**No. (row %)**	***P* value [a]**	**AOR (95% CI) [b]**	***P* value**
**Receipt of immunotherapy**					
No (neoadjuvant chemotherapy alone)	2641 (66.5)	1331 (33.5)	<.001	1 [Reference]	
Yes (neoadjuvant chemotherapy and immunotherapy)	2095 (57.2)	1567 (42.8)		1.45 (1.31-1.61)	<.001
**Age at diagnosis**					
Mean (SD)	56.0 (13.5)	52.9 (13.3)	<.001	0.83 (0.80-0.86) [c]	<.001
Median (IQR)	57.0 (46.0, 66.0)	53.0 (43.0, 63.0)	<.001	NA	
**Race/Ethnicity**					
Asian or Pacific Islander	232 (61.7)	144 (38.3)	.04	0.88 (0.70-1.11)	.29
Black	1104 (64.2)	617 (35.8)		0.90 (0.80-1.02)	.11
Hispanic	425 (57.4)	315 (42.6)		1.06 (0.89-1.25)	.54
White	2878 (62.1)	1759 (37.9)		1 [Reference]	
Other	110 (60.8)	71 (39.2)		1.05 (0.76-1.45)	.78
**Histologic type**					
Ductal	4276 (60.7)	2765 (39.3)	<.001	1 [Reference]	
Ductal and lobular	46 (75.4)	15 (24.6)		0.55 (0.28-1.06)	.07
Inflammatory breast cancer	141 (71.2)	57 (28.8)		0.84 (0.56-1.27)	.41
Lobular	61 (81.3)	14 (18.7)		0.72 (0.38-1.34)	.30
Metaplastic breast cancer	147 (83.1)	30 (16.9)		0.38 (0.25-0.59)	<.001
Other	78 (75.7)	25 (24.3)		0.65 (0.39-1.09)	.10
**Tumor grade**					
1	40 (85.1)	7 (14.9)	<.001	1 [Reference]	
2	857 (76.0)	270 (24.0)		1.42 (0.61-3.29)	.42
3	3763 (59.3)	2586 (40.7)		2.69 (1.17-6.18)	.02
**Charlson-Deyo Comorbidity Index**					
0	3843 (60.8)	2477 (39.2)	<.001	1 [Reference]	

1	635 (65.9)	329 (34.1)		0.90 (0.77-1.06)	.21
≥2	271 (73.1)	100 (27.0)		0.73 (0.56-0.94)	.02
**AJCC clinical T stage**					
cT2	3232 (60.5)	2109 (39.5)	<.001	1 [Reference]	
cT3	783 (68.8)	355 (31.2)		0.67 (0.58-0.78)	<.001
cT4	386 (70.4)	162 (29.6)		0.73 (0.56-0.93)	.01
**AJCC clinical nodal status**					
Negative (cN0)	2478 (60.6)	1613 (39.4)	<.001	1 [Reference]	
Positive (cN1+)	1917 (65.5)	1011 (34.5)		0.84 (0.75-0.93)	.001

Abbreviations: SD, standard deviation; IQR, interquartile range; No., number; AOR, adjusted odds ratio; CI, confidence interval; AJCC, American Joint Committee on Cancer.

Note: Other is a racial/ethnic group listed in the NCDB, which represents patients who were classified as Other by local cancer registries. The NCDB does not specifically define race/ethnicity classified into Other.

[a] *P* values were generated based on Student's t, Wilcoxon rank-sum, or Pearson's X^2 tests, as appropriate.

[b] This multivariable logistic regression model included all variables in the table, including receipt of immunotherapy, age at diagnosis, race/ethnicity, histologic type, tumor grade, and the Charlson-Deyo Comorbidity index.

[c] Age at diagnosis was considered a continuous variable and therefore, the corresponding AOR and 95% CI are listed per 10-year increase in age.

eTable 5. Assessment of Racial and Ethnic Differences in Pathologic Complete Response among Patients With Stage II-III, Triple-Negative Breast Cancer Who Received Immunotherapy and Neoadjuvant Chemotherapy in the 2021 National Cancer Database

	Pathologic complete response		Logistic regression			
	Did not achieve	Achieved	Model 1		Model 2	
Characteristic	(n=2095 [57.2%])	(n=1567 [42.8%])				
	No. (row %)	No. (row %)	AOR (95% CI)	*P* value	AOR (95% CI) [a]	*P* value
**Race/Ethnicity**						
Asian or Pacific Islander	121 (60.2)	80 (39.8)	0.76 (0.55-1.04)	.08	0.82 (0.53-1.26)	.37
Black	469 (59.5)	319 (40.5)	0.88 (0.74-1.06)	.17	0.92 (0.73-1.17)	.49
Hispanic	189 (52.8)	169 (47.2)	1 [Reference]		1 [Reference]	
White	1272 (57.1)	956 (42.9)	1.31 (0.83-2.05)	.25	1.09 (0.60-1.96)	.78
Other	44 (50.6)	43 (49.4)	0.86 (0.81-0.91)	<.001	0.84 (0.74-0.94)	.004
**Age at diagnosis**, per 10-year increase			0.76 (0.55-1.04)	.08	0.82 (0.53-1.26)	.37
**Histologic type**						
Ductal	1,874 (55.8)	1485 (44.2)	1 [Reference]		1 [Reference]	
Ductal and lobular	24 (75.0)	8 (25.0)	0.41 (0.16-1.03)	.06	0.38 (0.12-1.19)	.10
Inflammatory breast cancer	70 (63.6)	40 (36.4)	1.08 (0.64-1.83)	.77	0.99 (0.52-1.89)	.97
Lobular	27 (79.4)	7 (20.6)	0.65 (0.27-1.56)	.34	0.42 (0.14-1.26)	.12
Metaplastic breast cancer	69 (85.2)	12 (14.8)	0.28 (0.15-0.53)	<.001	0.37 (0.18-0.75)	.006
Other	31 (67.4)	15 (32.6)	0.72 (0.36-1.45)	.36	0.54 (0.21-1.39)	.20
**Tumor grade**						
1	15 (83.3)	3 (16.7)	1 [Reference]		1 [Reference]	
2	353 (69.8)	153 (30.2)	1.67 (0.45-6.19)	.45	1.01 (0.20-5.24)	.99
3	1691 (54.8)	1395 (45.2)	2.86 (0.78-10.5)	.11	1.75 (0.34-8.96)	.50
**Charlson-Deyo Comorbidity Index**						
0	1742 (56.4)	1347 (43.6)	1 [Reference]		1 [Reference]	
1	245 (58.8)	172 (41.3)	0.99 (0.79-1.24)	.92	0.99 (0.74-1.32)	.94
≥2	108 (69.2)	48 (30.8)	0.74 (0.51-1.09)	.12	0.85 (0.54-1.33)	.47
**AJCC clinical T stage**						
cT2	1346 (55.0)	1102 (45.0)	1 [Reference]		1 [Reference]	

cT3	400 (65.1)	214 (34.9)	0.66 (0.55-0.81)	<.001	0.72 (0.56-0.92)	.01
cT4	184 (65.9)	95 (34.1)	0.64 (0.45-0.91)	.01	0.83 (0.54-1.33)	.37
**AJCC clinical nodal status**						
Negative (cN0)	991 (54.8)	816 (45.2)	1 [Reference]		1 [Reference]	
Positive (cN1+)	935 (61.2)	593 (38.8)	0.83 (0.72-0.97)	.02	0.86 (0.71-1.25)	.11

Abbreviations: No., number; AOR, adjusted odds ratio; CI, confidence interval; AJCC, American Joint Committee on Cancer.

Note: Other is a racial/ethnic group listed in the NCDB, which represents patients who were classified as Other by local cancer registries. The NCDB does not specifically define race/ethnicity classified into Other.

[a] Additionally adjusted for percent no high school degree quartile, median household income quartile, type of health insurance, rural-urban area, and facility type.

eFigure 1. Kaplan-Meier Curves of Overall Survival among Patients With Stage IV, Triple-Negative Breast Cancer with Chemotherapy in the 2019-2021 National Cancer Database, by Immunotherapy Use

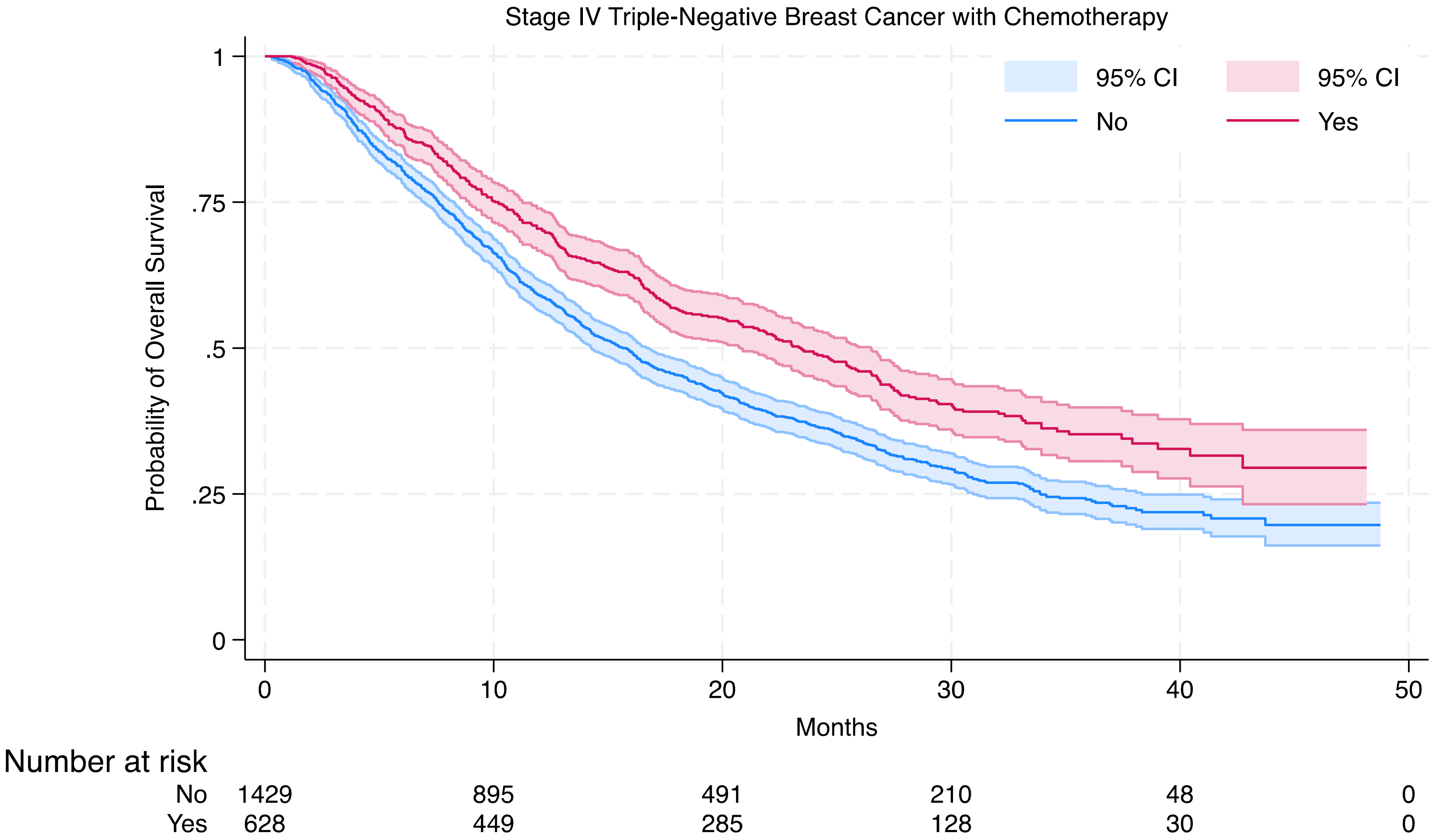

eTable 6. Kaplan-Meier Estimate of Median Overall Survival Time among Patients With Stage IV, Triple-Negative Breast Cancer with Chemotherapy in the 2019-2021 National Cancer Database

Characteristic	No. of patients	No. of events (%)	Median survival time in months [a] (95% CI)	*P* value [a]
**Overall**	2057	1297	17.3 (16.4-18.7)	
**Receipt of immunotherapy**				
No (chemotherapy alone)	1429	948 (66.3)	15.6 (14.3-16.8)	<.001
Yes (chemotherapy and immunotherapy)	628	349 (55.6)	23.4 (20.8-26.5)	
**Among patients who received immunotherapy**				
**Histologic type**				
Ductal	465	246 (52.9)	25.4 (22.3-27.8)	.05
Ductal and lobular	7	5 (71.4)	16.5 (8.5-.)	
Inflammatory breast cancer	81	53 (65.4)	16.5 (11.9-23.3)	
Lobular	16	11 (68.8)	18.4 (7.4-23.5)	
Metaplastic breast cancer	18	9 (50.0)	33.1 (13.2-.)	
Other	41	25 (61.0)	20.7 (8.3-.)	

Abbreviations: IQR, interquartile range, No., number; CI, confidence interval.
[a] *P* value was generated using the log-rank test.

eTable 7. Immunotherapy Use and Overall Survival among Patients With Stage IV, Triple-Negative Breast Cancer with Chemotherapy in the 2019-2021 National Cancer Database

	Cox proportional hazards regression			
**Characteristic**	**Model 1**		**Model 2**	
	**aHR (95% CI)**	***P* value**	**aHR (95% CI)** [a]	***P* value**
**Receipt of immunotherapy**				
Yes (chemotherapy and immunotherapy)	0.76 (0.66-0.87)	<.001	0.80 (0.68-0.94)	.006
No (chemotherapy alone)	1 [Reference]		1 [Reference]	
**Age at diagnosis**, per 10-year increase	1.02 (0.98-1.07)	.34	1.12 (1.03-1.22)	.007
**Race and ethnicity**				
Asian or Pacific Islander	0.90 (0.61-1.32)	.58	0.85 (0.53-1.37)	.50
Black	1.07 (0.93-1.22)	.34	1.03 (0.87-1.22)	.70
Hispanic	0.88 (0.68-1.13)	.30	0.74 (0.52-1.04)	.08
White	1 [Reference]		1 [Reference]	
Other	0.95 (0.60-1.50)	.81	1.39 (0.84-2.30)	.20
**Histologic type**				
Ductal	1 [Reference]		1 [Reference]	
Ductal and lobular	1.29 (0.78-2.13)	.32	1.31 (0.69-2.48)	.40
Inflammatory breast cancer	1.27 (1.07-1.51)	.006	1.31 (1.07-1.60)	.008
Lobular	1.08 (0.72-1.64)	.70	0.90 (0.55-1.45)	.66
Metaplastic breast cancer	0.65 (0.40-1.08)	.10	0.89 (0.53-1.50)	.67
Other	1.62 (1.19-2.21)	.002	1.52 (1.05-2.19)	.03
**Tumor grade**				
1	1 [Reference]		1 [Reference]	
2	2.14 (0.68-6.71)	.19	2.56 (0.63-10.43)	.19
3	2.14 (0.69-6.68)	.19	2.57 (0.64-10.38)	.19
**Charlson-Deyo Comorbidity Index**				
0	1 [Reference]		1 [Reference]	
1	1.12 (0.94-1.32)	.21	1.14 (0.94-1.38)	.18
≥2	1.52 (1.20-1.93)	.001	1.46 (1.12-1.92)	.01
**Type of health insurance**				

Medicaid	NA	NA	1.36 (1.10-1.69)	.004
Medicare	NA	NA	0.95 (0.77-1.18)	.65
Other government/unknown	NA	NA	1.17 (0.74-1.87)	.50
Private/managed care	NA	NA	1 [Reference]	
Uninsured	NA	NA	1.41 (1.01-1.96)	.04
**Type of cancer program**				
Academic/research	NA	NA	1 [Reference]	
Community	NA	NA	1.24 90.92-1.67)	.15
Comprehensive community	NA	NA	1.30 (1.10-1.53)	.002
Integrated network	NA	NA	1.08 (0.88-1.32)	.45

Abbreviations: No., number; aHR, adjusted hazard ratio; CI, confidence interval; NA, not applicable.

Note: Other is a racial/ethnic group listed in the NCDB, which represents patients who were classified as Other by local cancer registries. The NCDB does not specifically define race/ethnicity classified into Other.

[a] Additionally adjusted for percent no high school degree quartile, median household income quartile, and rural-urban area.
