# Supplementary material for: Trends and Disparities in the Use of Immunotherapy for Triple-Negative Breast Cancer in the US
Source: JAMA Netw Open. Author manuscript; Available in PMC 2025 Mar 25. (PMC11833518; doi:10.1001/jamanetworkopen.2024.60243)
Supplement: Supplemental Online Content 2 [file NIHMS2058962-supplement-Supplemental_Online_Content_2.pdf]

Data Sharing Statement

Title: Trends and Disparities in the Use of Immunotherapy for Triple-Negative Breast Cancer in the United States

Data

Data available: Yes

Data types: De-identified patient data

How to access data: Data for this analysis were obtained from the National Cancer Database (NCDB). Investigators associated with Commission on Cancer-accredited cancer programs can request the data by submitting a Participant User Data File (PUF) application to the American College of Surgeons via https://www.facs.org/quality-programs/cancer-programs/national-cancer-database.

Supporting Documents

Document types: None

Additional Information

Who can access the data: Anyone requesting the data

Types of analyses: Any purpose

Mechanisms of data availability: Requires a PUF application and a signed data use agreement according to the NCDB https://www.facs.org/quality-programs/cancer-programs/national-cancer-database/puf/
