# Supplementary material for: Trends and Disparities in the Use of Immunotherapy for Triple-Negative Breast Cancer in the US
Source: JAMA Netw Open. 2025 Feb 17;8(2):e2460243. doi: 10.1001/jamanetworkopen.2024.60243 (PMC11833518; doi:10.1001/jamanetworkopen.2024.60243)
Supplement: Supplementary file 2 [file jamanetwopen-e2460243-s002.pdf]

Data Sharing Statement

Freeman. Trends and Disparities in the Use of Immunotherapy for Triple-Negative Breast Cancer in the US. *JAMA Netw Open*. Published February 17, 2025. doi:10.1001/jamanetworkopen.2024.60243

Data
Data available: No

Additional Information
Explanation for why data not available: Data Data available: Yes Data types: De-identified patient data How to access data: Data for this analysis were obtained from the National Cancer Database (NCDB). Investigators associated with Commission on Cancer-accredited cancer programs can request the data by submitting a Participant User Data File (PUF) application to the American College of Surgeons via https://www.facs.org/quality-programs/cancer-programs/national-cancer-database. Supporting Documents Document types: None Additional Information Who can access the data: Anyone requesting the data Types of analyses: Any purpose Mechanisms of data availability: Requires a PUF application and a signed data use agreement according to the NCDB https://www.facs.org/quality-programs/cancer-programs/national-cancer-database/puf/
